# Prevalence and Patterns of Enteric Co-Infections Among Individuals Presenting with Cholera-like Diarrheal Disease During Seasonal Cholera Outbreaks

**DOI:** 10.3390/pathogens14121224

**Published:** 2025-11-30

**Authors:** Dhvani H. Kuntawala, Samuel Bosomprah, Bernard Phiri, Harriet Ng’ombe, Fraser Liswaniso, Mutinta Muchimba, Suwilanji Silwamba, Kennedy Chibesa, Bertha T. Nzangwa, Charlie C. Luchen, Innocent Mwape, Sekayi F. Tigere, Michelo Simuyandi, Nyuma Mbewe, Roma Chilengi, Amanda K. Debes, Nicholas R. Thomson, David A. Sack, Caroline C. Chisenga

**Affiliations:** 1 Basic Sciences and Immunology, Centre for Infectious Disease Research in Zambia, Lusaka 10101, Zambia; dhvani.kuntawala@cidrz.org (D.H.K.); samuel.bosomprah@cidrz.org (S.B.); bernard.phiri@cidrz.org (B.P.); harriet.ngombe@cidrz.org (H.N.); fraser.liswaniso@cidrz.org (F.L.); mutinta.muchimba@cidrz.org (M.M.); suwilanji.silwamba@cidrz.org (S.S.); kennedy.chibesa@cidrz.org (K.C.); bertha.nzangwa@cidrz.org (B.T.N.); chaluma.luchen@cidrz.org (C.C.L.); innokatebe@gmail.com (I.M.); sekayi.tigere@cidrz.org (S.F.T.); michelo.simuyandi@cidrz.org (M.S.); chilengir@yahoo.com (R.C.); 2 Department of Biostatistics, School of Public Health, University of Ghana, Accra P.O. Box LG13, Ghana; 3 Faculty of Science, Stellenbosch University, Private Bag X1, Matieland 7602, South Africa; 4 Department of Global Health, University Medical Center, Amsterdam Institute for Global Health and Development, University of Amsterdam, 1105 AZ Amsterdam, The Netherlands; 5 Zambia National Public Health Institute, Lusaka 10101, Zambia; nymbewe@gmail.com; 6 Center for Immunization Research, Johns Hopkins Bloomberg School of Public Health, Baltimore, MD 21205, USA; adebes1@jhu.edu (A.K.D.); dsack1@jhu.edu (D.A.S.); 7 Parasites and Microbes Programme, Wellcome Sanger Institute, Cambridge CB10 1SA, UK

**Keywords:** diarrheal, co-infections, cholera outbreak, HIV

## Abstract

Cholera remains a major public health challenge, and co-infections can complicate clinical outcomes. In a cross-sectional study, we investigated the prevalence and patterns of enteric co-infections during Zambia’s 2023–2024 cholera outbreak and evaluated their implications for disease severity. 240 suspected cholera patients were enrolled from five healthcare facilities in Lusaka. Stools were tested for 11 enteric pathogens using the Bosphore^®^ Gastroenteritis Panel Kit v2 on the QuantStudio 5 qPCR, with *Vibrio cholerae* confirmed by real-time PCR (quantitative PCR). Co-infections were highly prevalent, affecting 79.2% of participants. *Campylobacter* was the most frequently detected pathogen (70.0%), followed by Norovirus GI/GII (20.0%). Persons living with HIV were significantly more likely to present with co-infections than their counterparts (adjusted PR 1.27, 95% CI: 1.07–1.51; *p* = 0.008). Participants with confirmed *V. cholerae* + coinfections (N = 62) were less likely to developed moderate to severe disease compared to those with mono-infections (adjusted PR 0.59, 95% CI: 0.38–0.90; *p* = 0.014). These findings highlight the high prevalence and complexity of co-infections during cholera outbreaks, potentially contributing to antimicrobial resistance. They also highlight the need for targeted clinical management, particularly among persons living with HIV.

## 1. Introduction

Cholera, caused by *Vibrio cholerae* (*V. cholerae*), remains a significant global health challenge. In 2023, World Health Organization (WHO) recorded 535,321 cases and 4007 deaths worldwide, a 13% increase over 2022 [1,2]. This burden is concentrated in endemic regions; notably, sub-Saharan Africa observed large outbreaks in 2024. For example, from January to July 2024, WHO-AFRO documented 112,301 cases (1900 deaths, CFR 1.7%) across 14 countries, the bulk in Zimbabwe, Democratic Republic of Congo (DRC), Ethiopia, and Zambia [3].

Co-infections can markedly worsen cholera outcomes and undermine case management. For example, a recent Bangladesh study found that patients co-infected with toxigenic *E. coli* and *V. cholerae* experienced much more severe dehydration and vomiting, requiring intravenous fluids more often [4]. Likewise, surveys in Africa report that over half of diarrheal episodes in children involve multiple pathogens [5,6,7]. These mixed infections are not benign, but they intensify symptoms and complicate treatment, especially when coupled with comorbidities like HIV or malnutrition, potentially prolonging hospital stays. Moreover, co-infections create diagnostic ambiguity during outbreaks. With cholera symptoms overlapping many enteric diseases, clinicians often treat empirically. In a recent Syrian outbreak, 61% of patients with acute diarrhoea were given antibiotics, doxycycline or ciprofloxacin, despite uncertain aetiology [8]. Although guidelines reserve antibiotics for severe cholera, in practice they are frequently prescribed for all cases, a strategy that offers no benefit against viral or self-limiting pathogens and instead drives resistance. Indeed, strains of *V. cholerae* now exhibit extremely high resistance to older drugs (>96% resistance to co-trimoxazole in Ghana) [9] and rising resistance even to first- and second-line agents. In Zambia’s 2023–2024 epidemic, surveillance found emerging resistance to tetracycline and ciprofloxacin in Lusaka isolates (only ~84–82% remained susceptible) [10]. Targeted investigation of cholera co-infections in endemic regions is essential to close critical knowledge gaps, enhance diagnostic accuracy, promote rational antibiotic use, and ultimately improve patient outcomes while curbing antimicrobial resistance.

This study set out to determine the prevalence and patterns of enteric co-infections among individuals presenting with cholera-like diarrheal disease during Zambia’s 2023–2024 outbreak, and to assess their implications for clinical outcomes. Specifically, the study sought to identify the range of pathogens co-circulating with *V. cholerae*, examine demographic and clinical factors such as age and HIV status associated with co-infection risk, and evaluate the relationship between infection status and disease severity.

## 2. Materials and Methods

### 2.1. Study Design and Participants

We conducted a cross-sectional study, during the 2023–2024 cholera outbreak in Lusaka, Zambia, among children and adults who presented with rice water stool diarrhoea at five healthcare facilities namely: Levy Mwanawasa University Teaching General Hospital, George Health Centre, Matero Level One Hospital, Heroes Stadium Cholera Admission Centre), and Chipata Trades Mini Hospital. Participants were included if they tested positive for *V. cholerae* on rapid diagnostic test (RDTs) and in some cases based on clinical picture. HIV testing was mandatory at admission; therefore, HIV results were obtained from the Ministry of Health (MOH) dataset for all enrolled patients. Initial screening was conducted using a rapid HIV test.

Stool samples were collected in sterile containers and transported to the Basic Science and Immunology laboratory within 24 h at 4 °C. Upon arrival, samples were stored at −80 °C until analysis for cholera and multi-pathogen screening. Total nucleic acid extraction followed a previously described protocol [11]. Stool samples weighing approximately 150 mg were homogenized in SK 38 bead-beating tubes containing easyMAG^®^ Lysis Buffer (bioMérieux S.A., Marcy l’Etoile, France). The resulting homogenate was then centrifuged at 14,000 RPM for 2 min, and 200 μL of the supernatant was utilized for subsequent nucleic acid extraction using the Qiagen MinElute Kit (Qiagen, Hilden, Germany) according to the manufacturer’s instructions. Eluted nucleic acid was stored at −80 °C until ready for use.

### 2.2. Cholera Detection

Detection of *V. cholerae* was carried out using real-time PCR targeting the *ctxA* gene. Reactions were set up using the QuantiFast Pathogen RT-PCR +IC Kit (Qiagen, Germany), following the manufacturer’s instructions. DNA previously extracted from samples was thawed and added to a master mix containing the internal control, Taq polymerase, dNTPs, and gene-specific primers and probe for the *ctxA* gene (see Supplementary Table S1 for sequences). Amplification was performed on the 7500 Real-Time PCR System (Applied Biosystems, Foster City, CA, USA) using the following thermal cycling conditions: initial denaturation at 95 °C for 5 min, followed by 40 cycles of denaturation at 95 °C for 15 s and annealing/extension at 60 °C for 30 s. Samples were considered positive for *V. cholerae* when amplification of the *ctxA* gene occurred with a cycle threshold (Ct) value below 35 [12]. Each run included appropriate positive and negative controls to ensure assay validity and to monitor for potential contamination.

### 2.3. Multi-Pathogen Amplification and Detection of Other Enteric Pathogens

This study used a commercial Bosphore^®^ Gastroenteritis Panel Kit v2 (Anatolia Geneworks, Istanbul, Turkey), which detects 11 pathogens on the Real-time PCR Applied Biosystem Quantstudio 5 qPCR platform (Thermo Fisher Scientific, Waltham, MS, USA). The viral pathogens include astrovirus, rotavirus, norovirus G1 and GII, adenovirus, while the bacterial pathogens comprise *Clostridium difficile*, *Campylobacter* spp., *Salmonella* spp., Enteroinvasive *E. coli* (EIEC) and *Shigella* spp., verotoxigenic *E. coli* (VTEC) and *Yersinia enterocolitica.* Specifically, two master mix (Viral and Bacterial) [13] were prepared as follows:

For the viral preparation, 20 μL of the master mix (PCR Master Mix containing a reverse transcription mixture) was aliquoted into PCR tubes, followed by the addition of 5 μL of nucleic acid material (sample/positive or negative control). Similarly, for the bacterial preparation, 20 μL of the master mix was pipetted into the PCR tubes, followed by the addition of 5 μL of DNA (sample/positive or negative control) (Supplementary Table S2). All preparations adhered to strict aseptic techniques to prevent cross-contamination during the setup process. The qPCR thermocycler conditions were performed as described in Supplementary Table S3, and positive samples were determined using a cycle threshold (CT) value cutoff of 35, as previously established in a similar study [14] and as recommended by the manufacturer.

### 2.4. Statistical Analysis

Baseline characteristics were summarised using frequency and percentage for categorical variables while continuous variables were summarised using median and interquartile interval (IQI). The primary outcome was defined as proportion of participants with *V. cholerae* co-infection with other enteric pathogens. Using frequency and percentage, we summarised *V. cholerae* co-infection with single enteric pathogen and combination of pathogens. We used log-binomial generalized linear model to identify factors independently associated with prevalence of co-infection. In a secondary analysis, we explored the association of infection status (co-infection vs. *V. cholerae* alone) with disease severity. Disease severity was categorized as 0 = mild and 1 = moderate to severe. Treatment plan A corresponded to mild cases, while plans B and C were used for moderate to severe cases. All analyses were performed using Stata version 18.0 MP (StataCorp, College Station, TX, USA), and a two-sided *p*-value < 0.05 was considered statistically significant.

## 3. Results

### 3.1. Participants’ Flow and Background Characteristics

**Figure 1:**
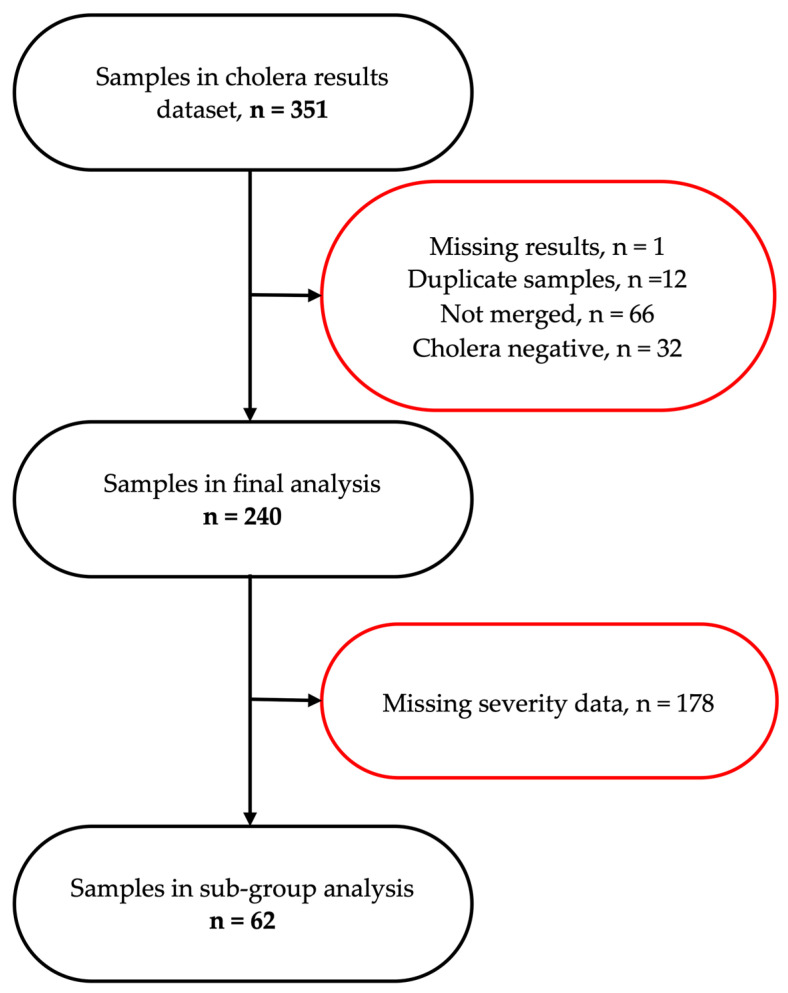
A schematic representation of participant flow through the study, detailing the selection process for stool samples included in the final and sub-group analyses.

A total of 351 participants were screened, of which 240 were included in the primary analysis (Figure 1). Among participants, 46.7% were male, 32.1% female, and 21.2% had missing sex data. The median age was 26 years (IQR: 14–38), with 19.6% under 15 years, 17.1% each in the 15–24 and 25–34 year groups, and 24.6% aged 35 years or older. Infection status varied significantly by health facility: 26 (10.8%) at Chipata, 62 (25.8%) at George, 54 (22.5%) at Heroes, 8 (3.3%) at Levy, and 90 (37.5%) at Matero. Regarding HIV status, 215 participants (89.6%) were HIV-negative and 19 (7.9%) were people living with HIV (PLWH) (Table 1). Notably, missing data were present for several key variables, including sex, age, HIV status, and cholera vaccination history.

### 3.2. Prevalence and Patterns of Enteric Co-Infections

**Figure 2:**
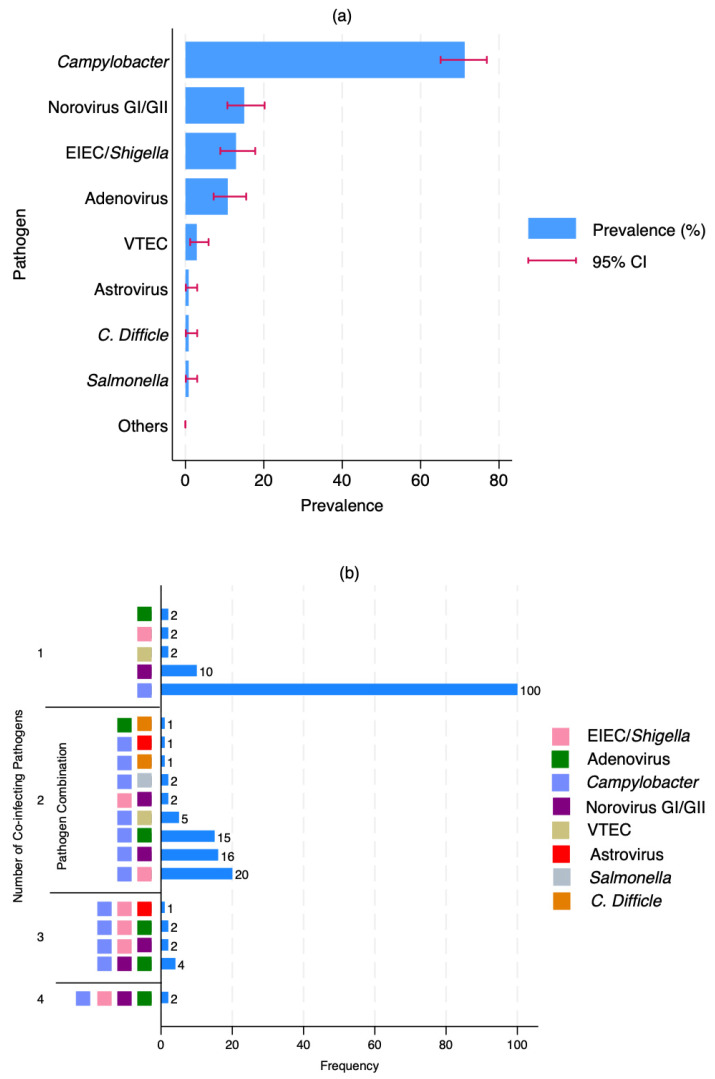
Prevalence and patterns of enteric co-infections. (**a**) prevalence of each pathogen plus *V. cholerae*. The horizontal bars represent the prevalence (%), and the red error bars indicate the 95% confidence intervals (CI); (**b**) patterns and combinations of pathogen co-infections plus *V. cholerae*. The bar chart shows the number of samples with 1 to 4 co-infecting pathogens and coloured squares indicate the specific pathogen(s) involved in each combination.

We assessed the prevalence and co-infection patterns within the study cohort. For this, we analysed pathogen-specific detection frequencies and their co-occurrence profiles (Figure 2). As shown in Figure 2a, we found the prevalence of *Campylobacter*, approaching 70%, followed by Norovirus GI/GII, with a considerably lower prevalence of approximately 15%. Moderate detection rates were also observed for EIEC/*Shigella* and Adenovirus, whereas other pathogens, including VTEC, Astrovirus, *Clostridioides difficile*, *Salmonella*, and those grouped as “Others,” were infrequently detected, each accounting for less than 5% of cases.

Co-infection analysis revealed that *Campylobacter* was not only the most common single pathogen (n = 100) but also the most frequently involved in co-infections (Figure 2b). Notably, we identified frequent co-infections involving *Campylobacter* and EIEC/*Shigella* (n = 20), *Campylobacter* and Norovirus GI/GII (n = 16), as well as *Campylobacter* and Adenovirus (n = 15). Less common co-infections included *Campylobacter* paired with VTEC, *C. difficile*, or *Salmonella*. Additionally, triple-pathogen co-infections involving *Campylobacter*, EIEC/*Shigella*, and Norovirus GI/GII were detected (n = 4). Although rare, we also observed two instances of quadruple infections comprising *Campylobacter*, EIEC/*Shigella*, Norovirus GI/GII, and Adenovirus.

### 3.3. Risk Factors of Co-Infection

Co-infection was identified in 79.2% (n = 190) of participants. Age and HIV status were significantly associated with co-infection (Table 2). Compared with children under 15 years, participants aged 15–24 and 25–34 years had a lower prevalence of co-infection (adjusted PR: 0.75; 95% CI: 0.58–0.96 and 0.80; 95% CI: 0.64–1.00, respectively). Conversely, PLWH were significantly more likely to have co-infection than HIV-negative participants (adjusted PR: 1.27; 95% CI: 1.07–1.51, *p* = 0.008).

### 3.4. Relationship Between Infection Status and Disease Severity

Of the 62 participants with severity data, 48 (77.4%) had co-infections and 14 (22.6%) had mono-infections as shown in Table 3. When comparing disease severity (Table 4), 71.4% of mono-infected patients presented with moderate-to-severe illness, compared to 47.9% of those with co-infections. After adjustment for sex, age group, and HIV status, co-infection was significantly associated with a lower risk of moderate-to-severe disease (adjusted PR: 0.59; 95% CI: 0.38–0.90, *p* = 0.014).

## 4. Discussion

This study investigated the prevalence and patterns of enteric co-infections among patients presenting with cholera-like diarrheal disease during Zambia’s 2023–2024 outbreak and assessed their implications for clinical outcomes. We found a high prevalence of enteric co-infections among individuals presenting with cholera-like diarrhoea during Zambia’s 2023–2024 outbreak. Nearly four out of five participants were co-infected, with *Campylobacter* and Norovirus GI/GII emerging as the most frequent co-pathogens alongside *V. cholerae*. PLWH status was strongly associated with co-infection risk, while individuals aged 15–34 years were less likely to be co-infected compared to children. Contrary to our initial expectations, co-infection was not associated with greater illness severity; instead, mono-infected participants were more likely to experience moderate-to-severe disease.

Among the co-infecting pathogens, *Campylobacter* was the most prevalent enteric pathogen among suspected cholera cases, detected in approximately 70% of cases, followed by Norovirus GI/GII (15%). This aligns with and vastly exceeds many reported prevalent rates particularly, in sub-Saharan Africa, where two systematic reviews found prevalence of 8.33% [15] and 9.9% [16], with individual country studies ranging from 1.7–62.7% [17]. The high burden of *Campylobacter* emphasizes its leading role in diarrheal disease and aligns with existing evidence that *Campylobacter* is a major cause of bacterial gastroenteritis worldwide [18,19]. However, our findings extend this understanding by quantifying its prevailing position in both single and multi-pathogen infections in this population. The prevalence for Norovirus GI/GII (15%) was similar to a study in South Africa (14.7%) [20], and Tanzania (11.44%) [21] and lesser when compared to 4.2% in Mozambique [22] and 9.9% in the Central African Republic [23]. We further demonstrate that co-infections were frequent, affecting nearly 80% of cases, and involved complex pathogen combinations such as Campylobacter with EIEC/*Shigella*, norovirus GI/GII, and Adenovirus. Results from Peru indicate a strong association between *Campylobacter* and *Shigella* infections and severe gastrointestinal disease, accounting for the majority of dysentery and severe diarrhoea cases [24]. Our findings indicate a higher co-infection rate than an earlier study from Bangladesh, which reported *V. cholerae–Campylobacter* co-infections in 4.5% of cases. However, a later study in the same region documented a substantially higher prevalence (40.7%) between 2016 and 2021 [25]. Notably, studies reporting co-infection conducted in Tanzania, also observed EIEC/*Shigella* significantly associated with diarrhoea both as mono infections and as coinfections [21,26]. While previous studies reported lower prominence of *Campylobacter* in co-infections, we observed its dominant role alongside both bacterial and viral pathogens, likely attributable to its immunomodulatory capacity, inducing inflammation and compromising host defences [27,28,29]. This contrasts with studies where viral pathogens primarily drive co-infections [30], highlighting complex enteric pathogen dynamics in this setting.

Host factors also appeared to influence the occurrence and outcomes of these infections in our study. We found that PLWH have a significantly higher risk of enteric co-infections compared to HIV-negative individuals, which is consistent with previous reports [31,32]. This demonstrates heightened susceptibility to enteric pathogens among immunocompromised individuals [33,34]. Age similarly played a significant role, reflecting the evolving immune competence and exposure risk across early life. Co-infections appear to be a particularly important mechanism underlying this age-related pattern, as young children in high-burden settings face greater pathogen exposure that directly interferes with oral vaccine effectiveness [35]. Ref. [36] identifies coinfection with other pathogens as a key host factor impacting cholera vaccine efficacy in children, while [37] describes how microbial overload on mucosal surfaces alters immune responses to oral vaccines. The intestinal environment of young children may be especially vulnerable, as [35] suggests that mucosal pathology secondary to infection interferes with vaccine responses and [38] identifies intestinal parasitic infections as important factors in vaccine hyporesponsiveness. This creates a concerning cycle where the children at highest risk for severe disease (those with the greatest pathogen exposure) are also least likely to benefit from vaccination protection [39]. While some studies conducted in sub-Saharan contexts show variation in co-infection prevalence by HIV status, possibly due to differences in antiretroviral therapy coverage and local pathogen ecology [40,41], the overall evidence indicates that weakened immunity is a major factor driving the heightened risk in this setting. Interestingly, disease severity in our cohort was not limited to co-infections. About 72% of mono-infected participants presented with moderate-severe symptoms, which contrasts with assumptions that co-infections invariably lead to worse clinical outcomes [42,43]. In a similar setting, Chisenga C.C and team found one-third of moderate-to-severe diarrhoea among children were attributable to *Shigella* and rotavirus in a cross-sectional study [11]. Evidence from other populations has shown similar trends, as well [26,44], suggesting mono-infections can be equally severe. However, our results differ from those of [45,46] showing increased severity with co-infections in different cohorts. One plausible explanation is that specific pathogens, such as *V. cholerae* or *Campylobacter*, primarily drive clinical manifestations, with co-circulating organisms contributing less to overall severity [47,48].

Hence, the presence of both bacterial and viral pathogens complicates treatment, as antibiotics are ineffective against viruses [49]. The observation that mono-infections can already present with moderate-severe symptoms supports the concept that disease severity may be more dependent on host factors, pathogen virulence, and individual immune responses rather than simply the number of co-infecting pathogens [50]. This diagnostic complexity becomes critical when differentiating between bacterial, viral, and mixed infections, often resulting in inappropriate antimicrobial use. Recently, Panda, P. K. [51] highlighted how viral infections misclassified as bacterial prompt unnecessary antibiotic therapy, accelerating AMR and increasing reliance on advanced antimicrobials. This diagnostic challenge is compounded in co-infection scenarios where [52] complex pathogen interactions complicate both disease progression and treatment protocols, making accurate diagnosis more difficult. It also [53] reveals that coinfecting pathogens can substantially impact resistance evolution through immune modulation and drug interactions [54]. This further represents a systematic issue where antibiotic use consistently exceeds actual bacterial coinfection incidence, suggesting that diagnostic failures at the clinical level have far-reaching implications for global AMR patterns.

We, however, recognized some limitations. The small sample size in our study likely contributed to the wide confidence intervals, which may have influenced by wide variability within the study population. Also, a substantial proportion of participants had missing data on key variables such as sex, age, HIV status, and vaccination history, potentially introducing bias. A larger cohort study would be ideal to measure the effect size of our outcome variable accurately. We also acknowledge the study relied on PCR evidence without any culture confirmed *Campylobacter* cases or a control group to differentiate between true infections and asymptomatic carriage. It would be important to incorporate culture-based methods and control populations to strengthen validity. Variability in surveillance data quality and possible underreporting further complicate efforts to accurately estimate the actual disease burden. The findings indicate the critical role of non-Vibrio pathogens in cholera-like diarrheal outbreaks and highlight the limitations of focusing only on *V. cholerae* alone in disease control efforts. Despite these limitations, to the best of our knowledge, this is the first study in Zambia to investigate the aetiology of diarrhoea during a seasonal cholera outbreak, highlighting the critical insights into the complex interplay between *V. cholerae* and other enteric pathogens.

Our findings emphasize the importance of considering co-infections during cholera outbreaks, particularly among PLWH who appear more susceptible. Clinicians should be aware that the presence of co-infections may influence disease presentation and severity, and integrating broader pathogen surveillance into outbreak management could refine case assessment and guide more targeted antimicrobial use. Understanding pathogen–pathogen and host–pathogen interactions may help clinicians anticipate complications and optimize treatment strategies. If further studies confirm these observations, public health authorities may need to incorporate systematic co-infection monitoring into cholera outbreak response protocols. This could inform antimicrobial stewardship programs and support policies that allocate resources for comprehensive pathogen surveillance. Moreover, policy frameworks might need to consider HIV status as a factor in outbreak preparedness and response planning, particularly in high-prevalence regions, to ensure interventions are equitable and effective. Thus, to inform public health responses, further investigation is necessary to explore the mechanisms behind the evident protective association of co-infections with reduced cholera severity. Such studies could identify novel targets for therapeutic intervention or preventive strategies. Broader investigations into co-infection patterns during outbreaks could also provide critical insights into disease dynamics and inform future outbreak modelling and awareness.

## 5. Conclusions

In conclusion, enteric co-infections were common during Zambia’s 2023–2024 cholera outbreak, with *Campylobacter* and Norovirus frequently co-circulating alongside *V. cholerae*. HIV positivity increased the risk of co-infection, while co-infected individuals experienced less severe infection compared to those with mono-infections. Enteric co-infections during cholera outbreaks may have a particularly pronounced impact among PLWH, influencing both disease risk and severity. These findings provide new insights into the epidemiology and clinical impact of co-infections in cholera outbreaks and highlight the complex interplay between pathogens and host factors. They highlight the importance of integrated diagnostic and surveillance strategies and point to the need for targeted interventions to optimize patient care, particularly among vulnerable populations during outbreak settings.

## Figures and Tables

**Table 1:** Baseline characteristics of participants.

Characteristic	Total N = 240
	n (% of Total)
**Sex**	
Male	112 (46.7)
Female	77 (32.1)
Missing	51 (21.2)
**Age (Years),** Median (IQI *)	26 (14–38)
**Age group (Years)**	
<15	47 (19.6)
15–24	41 (17.1)
25–34	41 (17.1)
35+	59 (24.6)
Missing	52 (21.7)
**Facility**	
Chipata	26 (10.8)
George	62 (25.8)
Heroes	54 (22.5)
Levy	8 (3.3)
Matero	90 (37.5)
**Vaccinated against cholera**	
No	18 (7.5)
Yes	4 (1.7)
Missing	218 (90.8)
**HIV Status**	
Negative	215 (89.6)
Positive	19 (7.9)
Missing	6 (2.5)

* Interquartile Interval.

**Table 2:** Risk factors of co-infection.

Characteristic	Co-Infection (n = 190, 79.2%)	*p*-Value	Unadjusted PR *	*p*-Value	Adjusted PR	*p*-Value
	n (% of Row Total)		Ratio (95% CI)		Ratio (95% CI)	
**Sex**						
Male	87/112 (77.7)	0.865 a	Reference	0.866		
Female	59/77 (76.6)		0.99 (0.84, 1.16)			
**Age group (Years)**						
<15	41/47 (87.2)	0.080 a	Reference	0.098	Reference	0.068
15–24	28/41 (68.3)		0.78 (0.62, 0.99)		0.75 (0.58, 0.96)	
25–34	29/41 (70.7)		0.81 (0.65, 1.02)		0.80 (0.64, 1.00)	
35+	49/59 (83.1)		0.95 (0.81, 1.12)		0.92 (0.78, 1.09)	
**Vaccinated against cholera**						
No	14/18 (77.8)	1.000 b	Reference	0.910		
Yes	3/4 (75.0)		0.96 (0.51, 1.81)			
**HIV Status**						
Negative	166/215 (77.2)	0.084 b	Reference	0.002	Reference	
Positive	18/19 (94.7)		1.23 (1.08, 1.40)		1.27 (1.07, 1.51)	0.008

* PR = Prevalence Ratio, a = Chi2, b = Fisher’s exact.

**Table 3:** Background Characteristics of Participants with severity data.

Characteristic	Total N = 62
	n (% of Total)
**Sex**	
Male	30 (48.4)
Female	32 (51.6)
**Age,** Median (IQI *)	27 (20–40)
**Age group**	
<15	1 (1.6)
15–24	9 (14.5)
25–34	15 (24.2)
35+	16 (25.8)
**Vaccinated against cholera**	
No	14 (22.6)
Yes	4 (6.5)
Missing	44 (71.0)
**HIV Status**	
Negative	53 (85.5)
Positive	9 (14.5)
**Infection status**	
Mono-infection	14 (22.6)
Co-infection	48 (77.4)

* Interquartile Interval.

**Table 4:** Association of co-infection and disease severity.

Characteristic	Moderate-to-Severe (n = 33, 53.2%)	*p*-Value	Unadjusted PR *	*p*-Value	Unadjusted PR *	*p*-Value
	n (% of Row Total)		Ratio (95% CI)		Ratio (95% CI)	
**Infection status**						
Mono-infection	10 (71.4)	0.121	Reference	0.080	Reference	0.014
Co-infection	23 (47.9)		0.67 (0.43, 1.05)		0.59 (0.38, 0.90)	

* PR = Prevalence Ratio, Ratios adjusted for sex, age group, and HIV status.

## Data Availability

All data generated and analysed during this study are included in the published manuscript and Supplementary Information Files. The data presented in this study are available upon reasonable request from the corresponding author. The CIDRZ Ethics and Compliance Committee is responsible for approving such request. To request data access, one must write to the Secretary to the Committee/Head of Research Operations, Hope Chinganya (Hope.Chinganya@cidrz.org). Dataset requests must include contact information, a research project title, a description of the proposed analysis, and the format in which it is expected. The requested data should only be used for the purposes related to the original research or study. The CIDRZ Ethics and Compliance Committee will normally review all data requests within 48–72 hours (Monday–Friday) and provide notification if access has been granted or additional project information is needed before access can be granted.

## Supplementary Materials

The following supporting information can be downloaded at: https://www.mdpi.com/article/10.3390/pathogens14121224/s1, Table S1: Sequences for Cholera detection; Table S2: PCR Master mix reagent conditions; Table S3: PCR Thermocycler Protocol.
