# Supplementary material for: Prevalence and Patterns of Enteric Co-Infections Among Individuals Presenting with Cholera-like Diarrheal Disease During Seasonal Cholera Outbreaks
Source: Pathogens. 2025 Nov 30;14(12):1224. doi: 10.3390/pathogens14121224 (PMC12736210; doi:10.3390/pathogens14121224)
Supplement: Supplementary file 1 [file pathogens-14-01224-s001.zip › pathogens-3986607-supplementary.pdf]

Supplementary Table S1 | Sequences for Cholera detection

**Target gene**	**Primer/Probe**	**Sequence (5'–3')**
*ctx*A	CtxA-F	GCATAGAGCTTGGAGGGAAGAG
	CtxA-R	CATCGATGATCTTGGAGCATTCA
	Probe	CATCATGCACCGCCG

Supplementary Table S2 | PCR Master mix reagent conditions

**Viral Master Mix**	**Volume**	**Bacterial Master Mix**	**Volume**
PCR Master Mix (Enzymes, dNTPs, Primers, Probes)	19.75 µl	PCR Master Mix (Enzymes, dNTPs, Primers, Probes)	20 µl
Reverse Transcription Mix	0.25 µl		
Internal Control	0.2 µl	Internal Control	0.2µl
Total nucleic Acid	5 µl	Total nucleic Acid	5 µl
(Negative/Positive Control)		(Negative/Positive Control)	
**Total Volume**	25 µl	**Total Volume**	25 µl

Supplementary Table S3 | PCR Thermocycler Protocol

**Condition**	**Temperature**	**Time**	**Cycles**
Reverse Transcription	50°C	15:00 min	Hold
Initial denaturation	95°C	05:00 min	
Denaturation	97°C	00:15 min	40
Annealing	60°C	01:00 min	
Hold	32°C	00:20 min	Hold
